# Antioxidants: A Hot Controversy Defused by Cool Semantics

**DOI:** 10.3390/antiox13101264

**Published:** 2024-10-18

**Authors:** Ahmad Yaman Abdin, Muhammad Jawad Nasim, Claus Jacob

**Affiliations:** Division of Bioorganic Chemistry, School of Pharmacy, Saarland University, D-66123 Saarbruecken, Germany; yaman.abdin@uni-saarland.de (A.Y.A.); jawad.nasim@uni-saarland.de (M.J.N.)

**Keywords:** antioxidants, complexity, language game, mechanistic causality, reductionism, semantics

## Abstract

Recent years have witnessed a rather controversial debate on what antioxidants are and how beneficial they may be in the context of human health. Despite a considerable increase in scientific evidence, the matter remains highly divisive as different pieces of new data seem to support both the pro- and the anti-antioxidant perspective. Here, we argue that the matter at the heart of this debate is not necessarily empirical but of semantics. Thus, the controversy cannot be resolved with the traditional tools of natural sciences and by the mere accumulation of new data. In fact, the term “antioxidants” has been part of the scientific language game for a few decades and is nowadays used differently in the context of different scientific disciplines active at different levels of scientific complexity. It, therefore, represents not a single expression but an entire family of words with distinctively different connotations and associations. The transcendent use of this expression from a basic to a more complex discipline, such as going from chemistry to physiology, is problematic as it assigns the term with connotations that are not corroborated empirically. This may lead to false claims and aspirations not warranted by empirical data. Initially, health claims may not even be indented, yet, on occasion, they are welcome for reasons other than scientific ones. To resolve this debate, one may need to refrain from using the term “antioxidants” in disciplines and contexts where its meaning is unclear, limit its use to disciplines where it is essential and beneficial, and, in any case, become more specific in such contexts where its use is warranted, for instance, in the case of “dietary antioxidants”.

## 1. Introduction

The rather unconventional title of this Special Issue, “Something is Rotten in the State of Redox”, is not simply an homage to William Shakespeare, Hamlet, and Denmark. Hydrogen sulfide (H_2_S), for instance, is clearly prominent within the biological redox community, not only thanks to its unique antioxidant properties and biological activity, but it also smells distinctively rotten, and the same applies to many other naturally occurring sulfur compounds. The title may, however, also be read in a more contentious manner, referring to some of the ongoing heated debates in the field of biological redox processes, which have become rather controversial and provocative during the last couple of years [1,2,3,4,5,6].

During this time, antioxidants have received significant scientific and public attention. Beyond various versatile applications in a range of industries, for instance, to safeguard food from oxidation or degradation, antioxidants have also been implicated in the protection from, and the fight against, numerous, often metabolic and age-related, diseases and disorders. The notion that a class of compounds can mitigate damage and prevent deterioration by neutralizing free radicals and other oxidative stressors is indeed attractive and promising across various disciplines, from chemistry and biology to nutrition and medicine. Eventually, this has moved the concept of antioxidants to the center of considerable controversy. Cherished by some and capitalized on by others, the notion of antioxidants has also been met with outright rejection. As one colleague recently put it:

“Except for the ‘elucidation of antioxidant mechanisms’, the scope [of such publications] contributes to a misleading and highly simplified concept of so-called (natural) antioxidants as ‘good and healthy’ agents vs. so-called oxidants as ‘bad and disease-associated’ molecules. I do not want to support this concept because I think it has extremely harmed the redox research community, maybe even beyond repair.”

(Personal communication of an eminent colleague in 2020 who prefers to remain anonymous.)

In fact, the debate of potential health benefits associated with antioxidants is not new and has been raging since the first publications in this field emerged in the 1980s [7,8,9,10,11]. It is a rather open dispute, encompassing colleagues and opinions from across the scientific spectrum, from chemistry to nutrition. Apart from their scientific relevance, antioxidants have found their way into trivial items such as teas sold in discounters and anti-aging cosmetics sold in drug stores. Therefore, the concept is not just “academic”; it also has considerable relevance outside the laboratory, in real life, society, and industry [12,13,14]. This brings not only scientists but also other vested interests, i.e., money, to the table, something we shall consider in more detail in Section 5. Indeed, talking about antioxidants has become a bit like Marmite^®^ (you either love it or hate it), with industry mostly liking it and some biologists hating some of it. It is, therefore, not unreasonable to claim that this matter has polarized and split the scientific community for years, maybe not quite “beyond repair”, yet still with a detrimental impact on joint interdisciplinary research [15].

At closer inspection, the “antioxidants controversy”, heated as it is these days, might not even be the outcome of conflicting scientific results, studies, or the interpretation of data [16]. It may, rather, be rooted in language and semantics and driven by the vested interests of the different players at the table. In other words, when using the expression “antioxidants”, we may not be referring to the same thing, and, perhaps quite intentionally, trigger different connotations and associations connected with this term.

In this perspective, we shall therefore question the ability of natural and life sciences to resolve this debate with the traditional tools available to them, as the problem at hand is more likely to be rooted in language and semantics. In such instances, we suggest the deployment of the iterative “Exeter method” proposed in 2001 and discuss the matter from the perspective of Theory of Science while involving epistemology, logic, and mechanistic and analytical philosophy [17,18]. In the specific matter of “antioxidants”, we shall advocate a linguistic turn and show that the word “antioxidants” is a homonym whose exact meaning depends on the scientific discipline and context in which it is used. Consequently, the term “antioxidants” has not one, finely delineated definition across science, but several quite different meanings and associations. Crossing from one discipline to another, especially from more fundamental to more complex and applied ones, requires us to constantly and carefully redefine the meaning of the word. Forgetting to exchange the definition of “antioxidants” to the local currency of that more complex discipline may accidentally load the term with connotations and associations not justified empirically, thus resulting in major misunderstandings and, indeed, controversies. We shall also argue that this conflicting use of the term—at least in part—is not just an unfortunate coincidence but rather the result of various vested interests that, these days, dominate and nudge the discourse into a specific direction.

## 2. Reductionism and Semantics: A Recipe for Controversy

**Figure 1:**
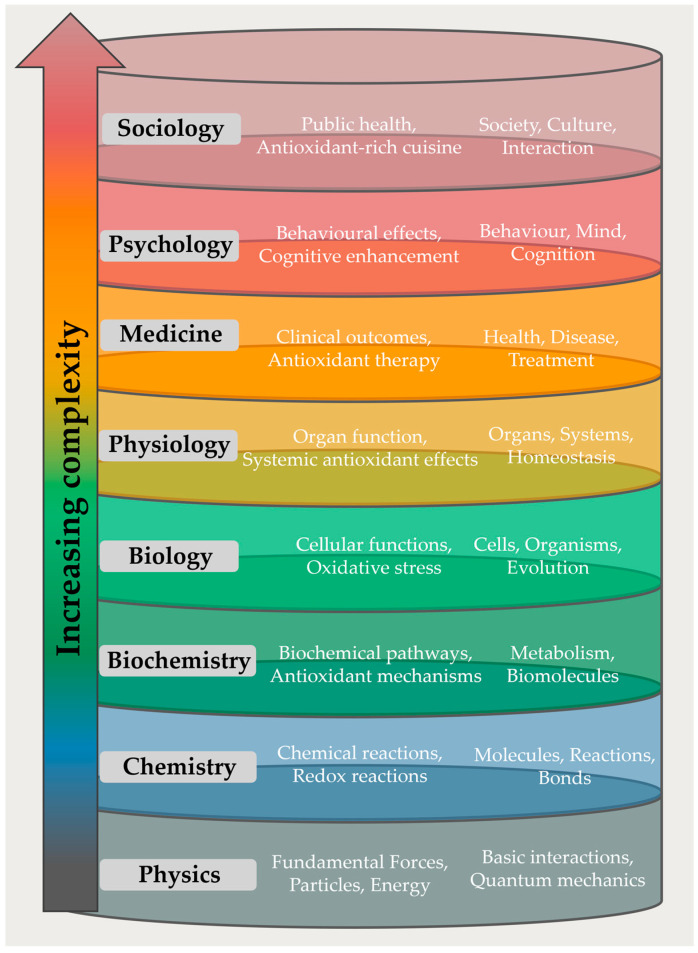
A version of the hierarchy of scientific disciplines. The figure demonstrates the increasing complexity from physics at the base to sociology at the top. Each layer represents a distinct scientific discipline with its associated keywords, emergent properties, and key elements.

The first issue one needs to consider in this debate is epistemological and relates to the reductionist method, which is dominant in modern science. This approach attempts to “dissect” the description of more complex scientific entities into simpler, more basic ones, for instance, by reducing (*sic*) the description of a living cell into a vast and admittedly complex network of chemical reactions, or by reducing our understanding of personal behavior to a finely conducted concert of neurotransmitters. This reductionist approach to science eventually results in a layered hierarchy of scientific disciplines, usually starting with physics as the most fundamental one at the bottom and proceeding upwards via chemistry, biochemistry, biology to physiology, medicine, psychology, sociology, and beyond (Figure 1). Each step upward adds complexity, yet also so-called “emergent” properties which are not found on the layer below, such as “life” and “thought” in the two examples just mentioned. Despite crossing different layers and, thus, disciplines, the reductionist perspective attempts to apply a uniform definition to scientific terms and aspires to standardize expectations about their functions or outcomes.

This approach of reducing phenomena to their simplest components has been extremely successful and foundational in scientific practice, allowing for the isolation of variables and understanding of fundamental principles in a controlled and predictable manner. Entire disciplines, such as cell biochemistry, systems biology, bioinformatics, personalized medicine, and psychiatry vastly depend on the concept of reductionism [19]. However, reductionism also poses inherent challenges when moving from basic disciplines such as physics and chemistry to more complex ones related to biology and psychology. This debate is not new and not even that alien to us: Consider the question of whether living cells or animals can be described as—or, whether they simply *are*—complex “machines”, as the French philosopher René Descartes claimed, or whether we can really reduce our thoughts to a dance of adrenaline, serotonin, dopamine, and a few other simple chemical molecules.

Several philosophers have therefore argued against reductionism. Thomas Kuhn, for instance, contended that science operates within paradigms, each having its own frameworks, tools, and methods, irreducible to one another [20]. Paul Feyerabend objected to the adoption of a single method or standard when discussing different scientific fields and considered it a limitation on creativity and the evolution of knowledge [21]. Both Kuhn and Feyerabend also emphasized the historical and sociological contexts of science and its practice. Science is laden with the influence and consequences of non-scientific factors and, therefore, cannot be fully understood through a reductionist lens, an issue we shall return to in Section 5. Others, such as Nancy Cartwright, remind us of the pitfalls of the idealizations and abstractions involved in reductionist scientific models and theories [22].

In many cases, and regardless of any “philosophy”, the results and conclusions of experiments conducted under controlled laboratory settings often prove to be meaningless in more complex systems. Failing to account for the distinct perspective and context of each different scientific explanation, i.e., plurality, when discussing complex phenomena introduces significant obstacles.

Besides many other such obstacles, reduction becomes especially controversial when trying to maintain consistent terminology across fields, a task frequently faced with semantic difficulties. In science, semantics refers to the meanings and interpretations of words and terms used within and across different disciplines. Each scientific field develops its own specialized language or jargon to address its unique questions and problems. This discipline-specific language may lead to significant semantic variations, even for terms that are slightly or superficially similar, and forms the basis of misunderstandings, as well as exquisite language jokes or puns.

In the movie Evolution, for instance, one of the answers given by a local university biology student to the question “What is a cell?” is, “A cell is 4 by 4 m, and my Uncle Joe is imprisoned in one”. This is, indeed, quite funny, and although no one outside Arizona would probably mix up a “prison cell” with a “living cell”, there are similar examples from the real world. The claim that selenium is “volatile”, for instance, caused a stir a few years ago in one of our postgraduate seminars. The inorganic chemists were on the barricades and close to their boiling points, the atmosphere in the room was, well, volatile, and the physiologists present insisted that selenium does indeed have rapid metabolism and excretion and that this had nothing to do with either evaporation or a specific psychological state of mind.

Some of these controversies can be resolved easily by avoiding terms that are either not defined or not warranted in a specific scientific discipline. The word “cell”, for instance, has no role in physics (apart perhaps from a battery), whereas the term “quark” may or may not be defined in biology as a subatomic particle, yet its practical use would be neglectable. Operationalists, such as Percy Bridgeman, would argue that you cannot isolate a quark with the tools of biology; thus, the term falls outside its realm, and pragmatists, such as Richard Rorty, would agree and, in a nutshell, assert that words or expressions which are de facto useless in a specific scientific discipline also have no role there [23,24].

As it happens, the volatile crowd in our example above was advised to use alternative, more discipline-specific expressions and to best avoid the word “volatile” in some contexts altogether. In the case of “cells”, scientists take another road and try to be more (discipline-) specific to avoid a potential mix-up by referring to composite terms such as “fuel cell”, “prison cell”, or “living cell”.

Semantics becomes especially problematic in situations when terms or expressions get closer in meaning. When similar terminology is used by scientists in similar fields, referring to a similar object, and used in a similar context, it triggers similar concepts and associations. Here, the precise, reductionist definitions used in one discipline do seemingly, yet not quite, align with the meanings assigned to the same terms in other fields. In such situations, the difference in what is meant and understood is so subtle that it creates a false sense of agreement, yet this is not really the case. At this point, reductionism and semantics become a true recipe for controversy!

This issue of agreement of language and terminology of different scientific disciplines in reductionism has not gone unnoticed and has been addressed at length by, for instance, Ernest Nagel, who has proposed a number of criteria for successful reduction, namely precise definitions of terms and sound bridge principles [25]. This approach is complex, and one example shall suffice to nail it down: To explain the role of activation energy in a chemical reaction, chemical kinetics quickly reduces to collision theory by focusing on energy and temperature. In this case, the reducing theory (collision theory) and the reduced theory (chemical kinetics) have precisely defined terms and are bridged through common concepts, such as energy and temperature.

Nagel’s approach aims to integrate knowledge from different disciplines and to create consistency in science. Despite its influence and utility, however, this method is perhaps best suited for the basic sciences, e.g., physics, and becomes quickly impractical, if not inapplicable, to more complex fields such as biology and those dealing with antioxidants.

Table 1 illustrates how common usage of the term “antioxidants” and its associations vary across different scientific disciplines, industries, and even public perception. In chemistry, for instance, the word antioxidant is uncommon and rarely used in daily routine. It does not even show up in most standard textbooks on general chemistry [26]. If used in chemistry, an antioxidant is primarily understood as an electron donor or a radical scavenger, focusing on its chemical properties and reactivity. In biochemistry, the emphasis shifts to its role in protecting biomolecules and inhibiting oxidative reactions, yet not necessarily with any reference to the “health” of the cell, tissue, or, indeed, an entire organism. Glutathione, for instance, is referred to as a cellular redox buffer [27]. This is wise, as oxidative stress also plays a role in many cellular functions, which is one of the reasons for the outrage of the colleague cited in the Introduction of this paper. Physiology and medicine, on the other hand, i.e., disciplines that deal with tissues and whole organisms, view antioxidants through the lens of their functional impact on health, such as reducing oxidative stress and preventing disease. Again, there is no clear “good” or “bad” there. Curiously, colleagues dealing with intact organisms often tend to ignore the chemistry of these molecules, leading to occasional claims that Zn^2+^ as a ”physiological antioxidant” is also an excellent electron donor or radical scavenger! In nutrition and, to some extent, also in cosmetics, antioxidants then turn into the “good and healthy agents” and ingredients fighting “bad and disease-associated molecules” such as free radicals and other oxidative stressors (see criticism on such attributions above). In nutrition and cosmetics, and in the industries associated with them, they are thus valued for potentially enhancing health and preventing (signs of) aging, albeit such claims are often controversial and legally questionable. It is, therefore, futile, and indeed dangerous, to assume that scientists from these different fields mean and refer to the same thing when talking about “antioxidants”.

The semantic variability associated with the term “antioxidants” is indeed considerable and not merely academic, as it has many practical implications—some scientific, others more dramatic. As just elucidated, Zn^2+^ ions cannot be called an antioxidant in chemistry as Zn^2+^ is redox-inactive under amenable conditions and unable to react with any oxidizing agents, ROS included. Yet, Zn^2+^ shows typical antioxidant properties at a physiological level where its addition to cells triggers the expression of thiol-containing antioxidant peptides, proteins, and enzymes, thus indirectly protecting them from oxidative stress while its absence causes oxidative damage. On the opposite side, hydrogen gas (H_2_) is an excellent reducing agent in chemistry, yet it is hardly bioavailable, may suffocate you upon inhalation, and no one would consider it in earnest as an antioxidant in medicine and start inhaling it!

## 3. Linguistic Turn

Indeed, once we inch closer to the exact definition of the term “antioxidants”, as reflected by Table 1, we may be surprised that (a) the term is present in the various terminologies of quite different disciplines, (b) the definition and associations with the term may differ quite substantially from discipline to discipline, (c) in most cases, an exact definition is notably missing, and (d) the term becomes richer in connotations and associations as one moves from less to more complex systems.

To avoid such controversies, it may be necessary to (re-)define the term “antioxidants” more sharply, and within each of the scientific disciplines the term is commonly used today. This may enable us to obtain a more relaxed and less controversial view of what an antioxidant is in chemistry, biochemistry, physiology, etc. While it is likely that the different definitions may still have much in common, they may not be quite the same and allow space for distinct differences and divergent associations and interpretations.

This issue of (scientific) language has been the center of the philosophy of the Austrian-British philosopher Ludwig Wittgenstein in his famous *Philosophical Investigations* (published in 1953) [28]. According to Wittgenstein, language is central; it not only serves for communication but also forms the basis for most of our activities, including the expression of emotions and making requests. As in the case of “antioxidants”, its terminology is not precisely defined and depends on the context and uses; thus, definitions, associations, and meanings are contextual and may differ from individual uses and users. Wittgenstein describes this as “language games”: activities with specific rules which, eventually, shape the meaning of words and form families with similar, yet not entirely identical, meanings. This “language game” has clearly been played out excessively with the word “antioxidants” since its introduction in the 1980s, and thousands of publications later, different users have used it differently, in different disciplines, at different times, and in different contexts. Thus, what we find for the term “antioxidants” today is an entire family, not just a single term, and this may also explain the many surprises when supposed antioxidants from in vitro studies fail to show any activity in animals or humans.

In comparison to expressions such as “cell” and “plasma”, the issues surrounding the uses of the word “antioxidants” are indeed more earnest. A “chemical antioxidant” may not be quite the same as a “cellular antioxidant” or a “dietary antioxidant”. Being more precise, such as in the term a “dietary antioxidant”, is therefore imperative and reflects the common distinction between a living cell, a fuel cell, and a prison cell; a volatile compound and a volatile personality; and physical plasma and blood plasma, as discussed at length above.

Alternatively, we may employ Ockham’s razor and avoid using the term “antioxidants” in certain disciplines altogether. In chemistry, for instance, one might argue it is not really fitting, i.e., it fails, as Percy Bridgeman would put it, to be operationally defined, meaningful, or, indeed, useful there. It could be replaced easily with better IUPAC-compatible terms, such as “reducing agent”, “electron donor”, or “radical scavenger”. These expressions are sharper, more specific, and, in any case, more meaningful within chemistry. They would also avoid endowing the term with associations it does not have, as well as mix-ups, such as considering the physiological and nutritional antioxidant Zn^2+^ an electron donor or radical scavenger.

We suggest a compromise between both strategies may be most fruitful, i.e., we should sharpen our razor and also use it. That is, to avoid this expression in disciplines where it has no basis, meaning, or real benefits and use composite terms such as “dietary antioxidant” in disciplines where using this term plays a beneficial role.

## 4. Misunderstandings

So far, we have focused on language. Yet, it is necessary to clean up a few misunderstandings that may creep in from overlooking the difference between how we describe things and their actual nature from an ontological point of view.

### 4.1. Mind the Ontological Gap!

Reductionism is not just about language. There is a difference between (scientifically) describing an object, process, or interaction, such as a human eating an apple rich in antioxidants, and the person, the apple, and its digestion “in itself”. There is also a difference between the definition, associations, and uses of the word “antioxidants” and the antioxidant “itself” as a substance, its properties, interactions, and, in essence, what it does in your body.

As discussed above from an epistemological perspective, reductionism in science is concerned with developing descriptions of subjects, objects, and interactions. The idea is to produce concepts, models, explanations, and predictions, then reduce them to a more basic form without dissecting the subject/object of the research itself. Since every scientific discipline has its own specialized view, those concepts, models, explanations, and predictions tend to differ. In our apple-eating example, views may range from the uptake and metabolism of chemicals in physiology, healthy eating in food sciences, sustainable nutrition with locally produced apples in ecology, to the psychology of an angry Newton eating his famous apple in protest to his classical physics being superseded by general relativity and the symbolic role in the story of Adam, Eve and the serpent in Judea-Christian theology. As disappointing as it may sound, science does not take a holistic, all-embracing view by posing and answering the question of what the person and apple are *per se*. This is still a matter of ontology, and it is unlikely that a uniform single science is to emerge, not least because it may be a very tedious and useless enterprise. Just imagine we get to the bottom of things and describe the consumption of this exquisite apple in terms of (sub-) atomic particles. *Bon appétit!*

Before one goes bananas about that and eats a frog, let us take a positive, albeit not positivistic, view on the fact that we as scientists are actually merely in the business of *constructing* knowledge, and that different builders construct different buildings from the same materials found in nature to provide us with housing and shops, hospitals, churches, and the odd post office. As much as you don’t buy stamps in a church, you also don’t ask about physiology in physics. As for the banana you ate this morning, a farmer may describe how the banana has been cultivated, harvested, and shipped to your home; a nutritionist may tell you about its composition, carbs, fat, protein, and fibers; a medic may inform you about its benefits for health; and Stormy Daniels may show you how to use it for some extra excitement in the White House. It is the same banana from an ontological point of view, of course, yet it turns into many different “stories” that have little to do with each other and have little causality or common language between them. Yet, each of them uses the term “banana”.

Therefore, let’s not be smarter than we are (or indeed can be) and claim that, regardless of what and how you say it, an antioxidant is “out there” as a real object, a substance that simply has all the properties and associations that the many different scientific disciplines from chemistry to nutrition have ascribed to it during the decades, and that these antioxidant properties simply emanate from this object and are therefore cast in stone and uniform across science. This view goes well beyond our capabilities, so please mind the ontological gap and stay within your discipline!

### 4.2. To Top-Down or to Bottom-Up?

**Figure 2:**
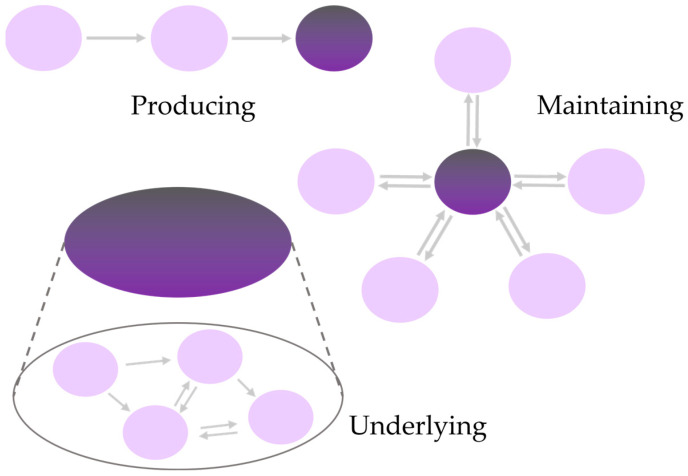
The three different types of mechanisms according to the new mechanist philosophy of science. A producing mechanism describes a cause-effect relationship within a defined level of complexity. A maintaining mechanism explains how a system’s stable state or continuous behavior is sustained. An underlying mechanism spans at least two levels of complexity, providing an inter-level, constitutive explanation. Depending on the focus of the analysis, a combination of these three types of mechanisms may be employed.

There is also the issue of causality. One may argue that, surely, we can dissect a banana, a frog, or indeed any other organism, apply light, electrons, and even atomic microscopes to obtain more details to get to the bottom of it! Although this may not be that fruitful. The water, sugars, proteins, fat, fibers, salts, etc. surely constitute the apple, banana, and frog, and thus their interactions on a lower level should also constitute their behavior, such as ripening or going to waste, at a higher one. As for antioxidants, a compound such as vitamin E or vitamin C, which reacts in vitro with H_2_O_2_, is likely to do so in a living cell or complex organism, and thus protects biomolecules and organisms from oxidative damage, right? Indeed, this is a question of mechanistic causes within and between layers of science (and the complexity of their objects), which has long been the focus of mechanistic philosophy and causality. Modern mechanistic philosophy, in general, and the Baumkuchen model, among others, distinguish different types of mechanisms, from a “producing mechanism” and “maintaining mechanism” within a specific layer to the “underlying mechanism”, which, in essence, transcends layers and is of special relevance here, as illustrated in Figure 2 [19,29,30,31].

At first sight, these mechanistic considerations may indeed tempt us to claim that “chemical antioxidants” can also be responsible for “health”, as their ability to remove oxidative stressors and free radicals contributes to our (biochemical) well-being in a wider sense [32,33]. This is, however, a trap, as transcending the layers “bottom-up” also means endowing the term with additional associations that may not have been present at the layer below. For instance, there are many agents with truly excellent antibiotic activity in cell culture, even in the nanomolar range. When added to an organism, however, no activity can be observed. Why? Simply because the “antibiotic” activity seen in the culture medium says nothing about the absorption, distribution, metabolism, and excretion (ADME) of the agent, i.e., the pharmacodynamics and pharmacokinetics. If one identifies an “antibiotic” in the Petri dish and then uses the same word in pharmacy or medicine, one literally charges this term with attributes and endows it with associations it did not have originally and which may not be warranted, as in the case of bleach. The latter does indeed kill most bacteria; yet calling it an exquisite antibiotic and drinking it against COVID-19, as a certain President suggested, not only ignores the fact that COVID-19 is caused by a virus, but it also shows that the meaning of and associations with the word “antibiotic” are more corpulent in medicine compared to the bog. From a more epistemological perspective, it illustrates that causality is to be discovered and defined “top-down”, for instance, by hunting for the underlying biochemical and chemical mechanism causing the activity of penicillin observed in living organisms, not vice versa. This is why it is called “underlying” and not “above-floating” in the first place, which brings us back to the bog and “antioxidants” again.

There may be an underlying causality for health benefits observed for drinking a strawberry shake or green tea rich in antioxidants, yet this benefit has to be observed first in the human population. Subsequently, the underlying mechanisms need to be determined “top-down”, i.e., from the perspective of physiology and medicine, and not by unjustified claims “bottom-up” from chemistry or in vitro biochemistry. Indeed, the health benefits may turn out to have nothing to do with the redox-active ingredients of the strawberry shake or green tea. Even if redox-active substances may turn out to be involved, they may exert their activities in a vastly different non-redox manner, not as antioxidants. They may, for instance, act as vitamins, such as vitamin E or vitamin C, or simply as supplements of a trace element, such as selenium. At the same time, certain excellent “chemical antioxidants” may be entirely bio-unavailable in an organism or arrive at their target at such low concentrations that their presence doesn’t even matter or is eclipsed by the activity of highly efficient enzyme-based cellular antioxidant defenses.

## 5. Foucault and Health Claims

This leaves us with the question of why the term “antioxidants” is experiencing controversial uses in science and society, whereas other related cross-discipline scientific expressions, such as “deoxynucleic acid”, do not. To be upfront on this issue, there may be many good and perfectly acceptable scientific reasons for this, such as incomplete and conflicting empirical data. However, this does not answer the question of why such a “misleading and highly simplified concept of so-called (natural) antioxidants as ‘good and healthy’ agents vs. so-called oxidants as ‘bad and disease-associated’ molecules” has actually formed and prevailed in the scientific literature for so long. Who decided to give molecules such judgmental attributes as “good and healthy” in the first instance?

An answer to this may be found in the philosophy of the French philosopher Michel Foucault, who, in essence, considers the societal impact on the “language games” discussed above [34]. According to Foucault, the discourse constructing knowledge through language and practices is embedded in and governed by social structures. As mentioned already, constructivists view the construction of knowledge as analogous and thus not that different from constructing a building, therefore highlighting the overarching role of the builders, their intentions, and also the societal factors influencing them, including architects, tools, equipment, suppliers, money, and external powers. According to Foucault, it is therefore essential to identify such discourses and their history, the relevant power relations at play, and the possible marginalizations of heretic views and dissent.

Throughout the history of science, we find ample examples that support this view, from aged luminaries dominating conferences with their (often outdated) views to “vested interests” of sponsors, from industry and governments to the military. It is indeed possible that, in such a setting, scientific discourse becomes skewed and marginalized. For instance, minority opinions, especially when held by junior scientists or the odd *persona non grata* sent to the wilderness rather than to conferences to present her/his data and interpretation thereof, may be ignored. Unbelievably, there are even rumors of sporadic cases of heretics not being given funding, laboratory space, or the opportunity to disseminate their discoveries, for instance in publications, as the funding agencies, selection committees, editorial boards, etc. are dominated by traditionalist builders of knowledge who are not really keen on anything *art nouveau*.

**Figure 3:**
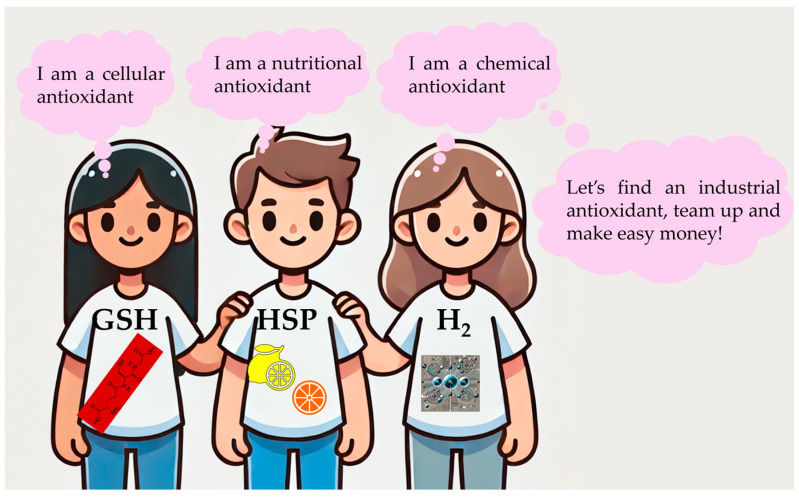
An illustration highlighting the varied perspectives of ”antioxidants” from different scientific fields. It exemplifies how discourse and self-interest can shape scientific narratives and public health claims. (The figure is partially generated by ChatGPT (GPT-4o), a language model developed by OpenAI, San Francisco, CA, USA).

Returning to the issue of “antioxidants”, we should therefore heed the advice of Foucault and briefly consider the rules and influencing factors of the discourse. At first, we may notice that the participants of this discourse come from various different scientific disciplines and thus may bring different scientific languages to the table. Consequently, they may not use the word “antioxidants” with the same meaning, eventually causing misunderstandings and unwarranted (health) claims, as already explained at length in the previous sections. Maybe there is a natural desire for such health claims in some disciplines, as they provide public exposure and a platform to reach out to society, industry and, to put it bluntly, money, as illustrated amusingly in Figure 3.

Indeed, antioxidants sell, in nutrition, as ingredients in foodstuffs and in nutritional supplements. The suspicion that some quarters are styling “antioxidants” as “good and healthy” is therefore not entirely unfounded. Quite amazingly, the elder and better looking of the authors of this perspective had the unique chance (twice) during the last ten years to get his face on a jar thanks to the antioxidants contained therein—one time for healthy strawberry jam rich in antioxidants, the other time for healthy green tea rich in antioxidants. Both offers were declined, not for scientific but for ethical reasons. Indeed, the construction of knowledge can be, on occasion, a sticky and dirty building site, always firmly embedded and bogged down in society [35]!

## 6. Keep Calm and Play Fair!

Our brief discussion on reductionism, semantics, language games, and the construction of knowledge has led us on a bumpy ride through difficult philosophical terrain. Eventually, it has shown that talking about “antioxidants” is controversial, predominantly because the expression is used differently and often imprecisely by different scientists active in different disciplines and at different layers of complexity. Taking the expression “antioxidants” from chemistry to more complex systems such as cells, tissues, and, eventually, humans automatically charge it with connotations and associations that are not really warranted. Similarly, the causality of underlying mechanisms must be determined “top-down” and not “bottom-up”, although “bottom-up” is perhaps easier and more desirable and is, therefore, often the reason behind unfounded “health claims” made for antioxidants from within the layers of chemistry, biology, and cell and tissue biology.

**Figure 4:**
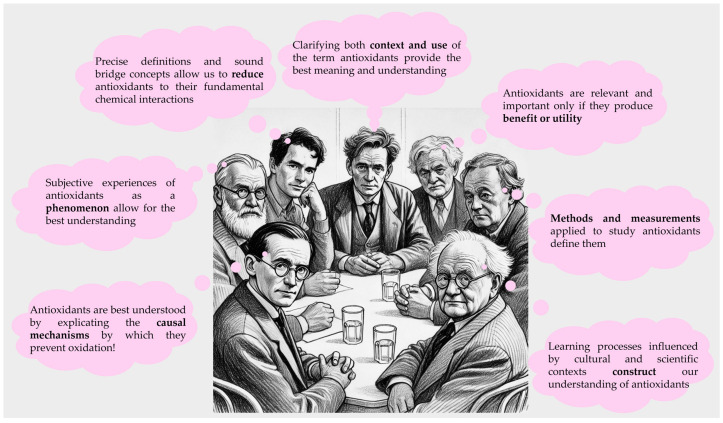
Different philosophical perspectives on the interpretation of the concept of antioxidants are presented from left to right: Carl Hempel would view antioxidants from the perspective of mechanisms and causality, Edmund Husserl would focus on phenomenology and subjective experience, Ernest Nagel would emphasize reductionism with precise definitions, Ludwig Wittgenstein would call for the clarification of use and context, Richard Rorty would inquire about their utility and benefit from a pragmatist viewpoint, Percy Williams Bridgman would define antioxidants through methods and measurements, and Jean Piaget would appreciate antioxidants as constructs of learning processes. (The figure is partially generated by ChatGPT, a language model developed by OpenAI).

To unwind (*sic*) and thus get rid of some of the rotten semantic stuff and bring this house into order, we may, therefore, heed the following pieces of advice from the many excellent philosophers of science we have called here in the witness box, as shown in Figure 4. Firstly, let’s accept that the term “antioxidants” is not one word with one definition and meaning; it stands for a family of different siblings who are closely related yet used differently in different scientific disciplines and at different layers of complexity. We may, therefore, better avoid the expression in basic scientific disciplines such as physics or chemistry, where it is de facto superfluous, and be more precise and careful in others by adding adjectives/attributes to form compositive terms, such as “dietary antioxidant” to distinguish one use from another. Playing this language game right may circumnavigate the most difficult issues. In addition, we should also stop from (un)willingly associating attributes such as “healthy” and “good” to such molecules, as this is not only a simplification but also outside the realm of the sciences making such claims. Indeed, the motivation behind such claims by non-scientists participating in this discourse needs to be spelled out clearly, as, on occasion, “money talks” in the form of funding and other incentives, yet this should not predominate or even drown the voice of science in the scientific debate.

Constructing the building of science is indeed a Texas job; it sometimes makes you sweat and thirsty for more (than you’ve got). So please play the language game fairly, and, in any case, don’t get stoned by the philosopher’s stone. Semantics is not everything, yet, surely, it helps!

## Figures and Tables

**Table 1:** The differences in terms associated with “antioxidants” based on context.

Context	Terms associated with “antioxidants”
Chemistry	Electron donor, radical scavenger, reducing agent
Biochemistry	Cellular redox buffer, protector of biomolecules, inhibitor of oxidation
Physiology	Oxygen species (ROS) neutralizer, cellular protector, homeostasis maintainer
Pharmacy	Oxidative degradation preventer, medication stabilizer, therapeutic agent
Medicine	Oxidative stress reducer, disease preventive, therapeutic agent
Nutrition	Food preservative, oxidation inhibitor, health-promoting compound
Cosmetics	Skin protector, anti-aging agent, environmental stressor shield
Industry	Oxidation preventer, material stabilizer, durability enhancer

## Data Availability

No new data was created.
